# Does Deep Squat Quality Affect the Propulsion of Baseball Throwing?

**DOI:** 10.3390/bioengineering11030248

**Published:** 2024-03-02

**Authors:** Wei-Hsuan Lin, Tsung-Yu Huang, Shu-Wei Chen, Joseph Hamill, Jung-Tang Kung, Wen-Tzu Tang

**Affiliations:** 1 Graduate Institute of Athletics and Coaching Science, National Taiwan Sport University, Taoyuan 333325, Taiwan; weixuanlin.terry@gmail.com (W.-H.L.); a3017927@gmail.com (T.-Y.H.); hsuwei0922@gmail.com (S.-W.C.); 2 Department of Kinesiology, University of Massachusetts Amherst, Amherst, MA 01003, USA; jhamill@kin.umass.edu; 3 Department of Sport Training Science-Balls, National Taiwan Sport University, Taoyuan 333325, Taiwan; kung@ntsu.edu.tw

**Keywords:** ground reaction force, joint mobility, movement assess, sports performance, training

## Abstract

This study investigates the influence of the quality of the “deep squat” movement, adapted from the Functional Movement Screen (FMS) system, on the lower extremity movement pattern during baseball throwing, and its potential impact on throwing performance and propulsion efficiency. Twenty-two baseball players were recruited and categorized into two groups: 13 in the high-score squat group (HSS) and 9 in the low-score squat group (LSS), based on their deep squat screening results. This research explored disparities in ball velocity, propulsion efficiency, propulsion ground reaction force (GRF) characteristics, and throwing kinematics between these two groups. The findings revealed no significant difference in ball velocity between the groups. However, the LSS group demonstrated a lower propulsion GRF efficiency (*p* < 0.030, ES = 0.46), along with a higher vertical peak GRF (*p* < 0.002, ES = 0.66). In the pivot leg, the HSS group exhibited significantly lower impulse forces in the Impulse Fresultant (*p* < 0.035, ES = 0.45), throwing direction (*p* < 0.049, ES = 0.42), and vertical direction (*p* < 0.048, ES = 0.42). Additionally, the contribution to the ball velocity of the pivot leg was significantly greater in the HSS group, along with significantly better efficiency in Impulse Fresultant (*p* < 0.035, ES = 0.45), throwing direction (*p* < 0.053, ES = 0.41), and vertical direction (*p* < 0.032, ES = 0.46). In the leading leg, the HSS group demonstrated significantly lower impulse forces in the Impulse Fresultant (*p* < 0.001, ES = 0.69), throwing direction (*p* < 0.007, ES = 0.58), and vertical direction (*p* < 0.001, ES = 0.70). Moreover, the contribution to the ball velocity of the leading leg was significantly greater in the HSS group, accompanied by significantly better efficiency in Impulse Fresultant (*p* < 0.003, ES = 0.63), throwing direction (*p* < 0.005, ES = 0.60), and vertical direction (*p* < 0.021, ES = 0.49). In conclusion, this study suggests that squat screening is a valuable tool for assessing propulsion efficiency. Coaches and trainers should be mindful of players with low squat quality but high throwing performance, as they may face increased impact and injury risks in the future.

## 1. Introduction

Baseball throwing is composed of multiple continuous movements from the lower to the upper limbs requiring coordination among all the joints [1,2]. Throughout the entire throwing process, muscle movement must be smooth and coordinated to improve velocity and accuracy [3]. Previous research has mainly focused on the throwing motion of the upper extremity, but overhead throwing requires coordination among joints, and the joints of the lower extremity contribute to the throwing motion [2,4,5]. The player’s feet are in a closed kinetic chain when throwing and the ground reaction force is transmitted from the lower limbs to the upper limbs through the pelvis and trunk [6,7]. In baseball pitching, the pivot leg generates forward body momentum towards home plate, while the lead leg, upon ground contact, acts as a braking mechanism, attenuating the forward momentum and facilitating kinetic energy transfer from the rotating trunk to the hands [2,5,8,9,10,11]. A previous study indicated that the peak GRF value and the impulse from the lower limbs to the upper limbs are important factors in transferring energy [10,12]. A study reported that a high-velocity group of athletes showed knee extension when approaching the ball release, whereas a low-velocity group showed a variety of knee movement patterns involving less knee extension and more knee flexion [13]. However, there are no studies on the impact of lower limb mobility on lower limb force.

If athletes lack a functional sports model, they will have a negative performance, efficiency, and skill level. Due to the advancements in sports science, the conventional routine assessment has been replaced by the assessment of athletes’ current physical state and training effect, and then the use of the assessment results as a reference to help athletes adjust their training menus. Sports science has suggested that the evaluation of the training process is increasingly important [14]. Assessing athletes’ daily routines can help athletes adjust their training content to improve athletic performance and achieve their goals.

The Functional Movement Screen (FMS) has been widely used to evaluate athletes [15,16,17]. Consisting of seven movement tests, the FMS quickly identifies compensatory movement patterns within the kinetic chain by screening bilateral limb asymmetry, mobility, and stability dysfunction [14,18]. Among the seven movements, the overhead squat has attracted considerable attention; the FMS design has even included a method for assessing the quality of overhead squats [15]. An overhead squat requires coordination among hip, knee, and ankle joints as well as excellent mobility throughout the kinetic chain [17]. Fitness coaches in Major League Baseball have also emphasized the importance of squats, and have used overhead squats to test the lower-limb flexibility of players [19]. Lower-limb movement is crucial in baseball throwing. Because the overhead squat test of the FMS measures coordination among the hip, knee, and ankle joints, as well as the flexibility of the kinetic chain, the FMS was applied to evaluate baseball players’ lower limb movement in this study. So far, no studies have examined the correlation between FMS overhead squat performance and the lower limbs’ biomechanical characteristics during overhead baseball throws.

Therefore, the purpose of this study was to use the FMS overhead squat test to investigate the potential correlation between baseball players’ lower-limb flexibility and their overhead throwing movement, lower extremity biomechanical characteristics, and throwing performance. We hypothesized that individuals with higher scores in the squat test would exhibit greater sagittal plane joint mobility, greater ground reaction forces, faster ball speeds, and improved propulsion efficiency. The findings from this study can offer valuable insights to baseball coaches and trainers, aiding them in designing effective technical and physical training routines.

## 2. Materials and Methods

The participants in the current study consisted of twenty-two elite college baseball players, who played in the Taiwanese College Division I League and won the National College Cup championship last year. The thirteen participants who performed full scores were in the high-score squat group (HSS; height: 179.69 ± 6.55 cm, body weight: 84.54 ± 14.77 kg, age: 21.08 ± 1.38 years, years of experience: 10.77 ± 2.20 years, throwing speed: 30.64 ± 3.33 m/s) and the remaining nine were in the low-score squat group (LSS; height: 181.11 ± 8.27 cm, body weight: 84.11 ± 18.11 kg, age: 21.11 ± 1.05 years; years of experience: 9.44 ± 2.26 years, throwing speed: 29.57 ± 2.6 m/s). To determine the sample size, a power analysis was conducted using G*Power 3.1, a total sample size of 22 participants would be sufficient to detect differences using the Mann–Whitney U-Test (effect size, d = 0.8; significance level, α = 0.05; power, 0.7). The inclusion criteria were that participants had undergone at least seven years of formal baseball training, five days a week for at least three hours a day. The exclusion criteria were that participants with injuries that could affect routine training or competition within the past 6 months were excluded from this study. The proposal for this study was approved by the Institutional Review Board of Fu Jen Catholic University, Taiwan (C103105). The participants were informed of the benefits and risks of the experiment before signing an informed consent form approved by the institution to participate in the study.

### 2.1. Procedures

**Figure 1:**
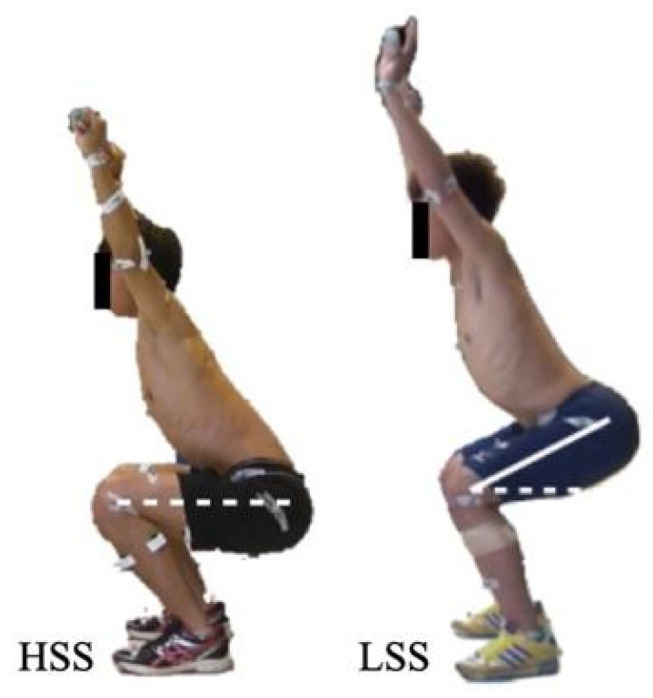
High-score squat group (HSS) and low-score squat group (LSS).

Functional movements were assessed using the FMS screening tool [14]. Two examiners, who attended the FMS course and were certified to administer the tool, assessed the participants. After receiving instructions for the FMS deep squat, participants performed three deep squats, which were evaluated simultaneously by both examiners. The assessment scores were consistent. The movements included ensuring that the upper torso was parallel with the tibia or toward vertical, the femur was below horizontal, the knees were aligned over the feet, and the dowel was aligned over the feet (Figure 1). They were reminded of the standard procedure before each deep squat. Participants who could complete all movements were classified into the HSS, while those unable to complete any of the movements were classified into the LSS.

Throwing movements were captured using a motion analysis system (Motion Analysis Corporation, Santa Rosa, CA, USA) at a sampling rate of 250 Hz. Two force plates were set horizontally to collect throwing data, a force plate recording the ground reaction force of the pivot leg (AMTI BP400600, 400 mm × 600 mm), and a force plate recording the ground reaction force of the lead leg (AMTI BP600900, 600 mm × 900 mm) (AMTI Inc., Watertown, MA, USA). Data sampling was accomplished at a rate of 1000 Hz [5].

A total of 43 reflective markers were adhered to specific landmarks to define the body segments and joint centers, following the reflective marker placement [5]. Initial calibration was collected to define the local coordinate systems of the body segments. During the experiment, the participants were asked to perform five throws with maximum effort. The data from the fastest trial were used for the analysis.

**Figure 2:**
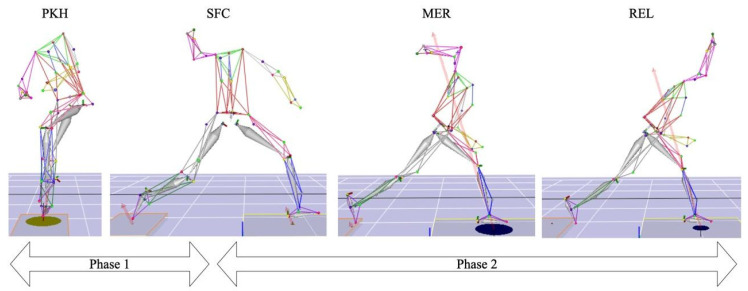
The definition of throwing phases.

To analyze the GRF of throwing movement, this study referred to the various phases of throwing [9]. The throwing movement was divided into two phases (Figure 2): The pivot leg phase (from the knee of the lead leg reaching its highest point to the lead leg touching the ground), and the lead leg phase (from the lead leg touching the ground to the ball releasing point). The direction definition of the force plate was based on the force plate coordinate system (positive facing the home plate, negative facing the second base). The front and rear (X direction) were set as the ball throwing direction, the inside and outside direction (Y direction) as the positive facing first base, the negative facing third base, and the vertical direction (Z direction) as the positive facing downward and the negative facing upward. The resultant ground reaction force was calculated from the sum of the forces in the three directions. The definition of the throwing phases is presented in Figure 2. The time of the pivot leg and lead leg are represented as a percentage in the two phases. In Phase 1, 0% corresponds to the lead leg at its peak knee height, and 100% corresponds to the lead leg’s instant of stride foot contact. In Phase 2, 0% corresponds to the lead leg’s instant of stride foot contact, and 100% corresponds to the ball release. Throwing speed was measured using a sports radar (The JUGS Gun Sport Radar Part No. R2050, JUGS Sports, Tualatin, OR, USA).

All participants wore sports shoes instead of wearing spiked shoes, as they normally would, due to the use of the force plate. The experimental space of this research was an indoor laboratory, hence the throwing distance for the experiment was 7.5 m instead of the regular 18.44 m [5]. After personal demographics (age, height, weight, medical history, and health status) were recorded, participants were asked to warm up using their personal routine. Participants were then asked to throw three strikes with maximum effort, from which the data of the fastest throw were used for the analysis.

### 2.2. Data Analysis

The 3D coordinates and the GRF were synchronized using Cortex v.1.1.4 software. Marker position data were filtered using a fourth-order Butterworth low-pass filter with a cut-off frequency of 13.4 Hz [20]. All temporal variables are presented as a percentage of relative time, where the timing of the GRF for the pivot leg was normalized and analyzed on a 0–100 scale during the stride phase (from the peak knee height of the lead leg ground contact). The timing of the GRF for the lead leg was analyzed on a 0–100 scale during phase 1 (from lead leg ground contact to ball release). The anterior, vertical, and resultant GRF variables for the pivot leg (including the peak value, peak timing, and the impulse of GRF during the stride phase) and the anterior, vertical, and resultant GRF variables for the stride leg (including the peak value, peak timing, and the impulse of the GRF during the arm cocking/acceleration phases) were analyzed. The ground reaction force data were normalized by dividing by the body mass of each participant. When analyzing the data from lead leg ground contact to ball release, we calculated the projection angle, maximum angle, minimum angle, and maximum angular displacement of hip flexion, knee extension, and ankle dorsiflexion during the pivot foot’s throwing motion. The ball speed efficiency was quantified by the ratio of ball speed to the ground reaction force (GRF) or the corresponding impulse.

### 2.3. Statistical Analyses

A statistical analysis of the variables for the GRF and lower-limb kinematics was conducted using SPSS 20.0 (International Business Machines Corporation, Armonk, NY, USA). The Kolmogorov–Smirnov test was used to analyze the data’s normality. Moreover, the data were not normally distributed, so we used a Mann–Whitney test. A Mann–Whitney U-Test was employed to examine the differences between the HSS group and the LSS group. Additionally, in order to make a statement about the effect size (ES) in the Mann–Whitney U-Test, we calculated the ES with the standardized test statistic z and the number of pairs n. In general, an ES less than 0.3 was a small effect. An effect size r between 0.3 and 0.5 was a medium effect. An effect size r greater than 0.5 was a large effect [21]. All data were presented as means ± standard deviation and statistical significance was determined at *p* < 0.05.

## 3. Results

Table 1 presents the peak GRF, and its efficiency (ball velocity/peak GRF), of the throwing motion in this study. The peak vertical direction of the GRF (*p* < 0.030, ES = 0.46) and its contribution to the ball velocity in the lead leg were significantly greater in the HSS group (*p* < 0.002, ES = 0.66) than in the LSS group, whereas no significant differences (*p* > 0.05) were observed in either the force in other directions or the pivot leg.

Table 2 presents the time when the ground reaction force reached its peak. No significant differences were detected between the HSS and the LSS groups for the GRF of the pivot leg and leading leg in all directions (*p* > 0.05).

Table 3 presents the impulse components and efficiency of both the pivot leg and lead leg. In the pivot leg, the HSS group exhibited significantly lower impulse forces in the Impulse F_resultant_ (*p* < 0.035, ES = 0.45), throwing direction (*p* < 0.049, ES = 0.42), and vertical direction (*p* < 0.048, ES = 0.42). Additionally, the contribution to the ball velocity of the pivot leg was significantly greater in the HSS group, along with significantly better efficiency in Impulse F_resultant_ (*p* < 0.035, ES = 0.45), throwing direction (*p* < 0.053, ES = 0.41), and vertical direction (*p* < 0.032, ES = 0.46). In the leading leg, the HSS group demonstrated significantly lower impulse forces in the Impulse F_resultant_ (*p* < 0.001, ES = 0.69), throwing direction (*p* < 0.007, ES = 0.58), and vertical direction (*p* < 0.001, ES = 0.70). Moreover, the contribution to the ball velocity of the leading leg was significantly greater in the HSS group, accompanied by significantly better efficiency in Impulse F_resultant_ (*p* < 0.003, ES = 0.63), throwing direction (*p* < 0.005, ES = 0.60), and vertical direction (*p* < 0.021, ES = 0.49).

The two groups exhibited no significant differences in the maximum angle, minimum angle, or maximum angular displacement of hip flexion, knee extension, and ankle dorsiflexion (*p* > 0.05) when throwing the baseball (Table 4).

## 4. Discussion

The aim of this study was to verify that poor squat quality can adversely affect performance. We found that, although the peak GRF was higher in the LSS group, the efficiency in the vertical direction of the lead leg was higher in the HSS group. Furthermore, no difference in ball velocity was shown between the HSS and LSS groups. A plausible explanation is that the elite players in the LSS group can perform on the same level in ball velocity as the HSS group to maintain the competitive level since the participants of both groups were all recruited from Division I. Previous studies showed that the high peak vertical and resultant GRF of the lead leg can contribute to ball velocity [2,8,9]. However, our studies showed that the LSS group showed a higher vertical propulsion force but with a lower efficiency, implying that the LLS must apply a greater peak GRF to maintain the same level of ball speed performance due to the low propulsion and kinetic chain efficiency.

In previous studies, there has been a contradiction between the effect on performance due to deficits or poor-quality functional movements [22,23,24]. The results of this study were able to provide a possible explanation for the contradiction and, thus, provide the first direct evidence that poor functional movement quality can affect progressive movement performance (such as propulsion efficiency). Pitchers appear to compensate for propulsion with a greater muscle load to maintain the same level of terminal performance (like ball speed). This suggestion also corresponds to previous studies showing that the inability to perform squats is commonly attributed to the physical limitations of closed kinetic chain dorsiflexion in the ankle, or thoracic spine extension [14]. Because baseball players throw with unilateral strength for long periods of time, this can cause differences in mobility between the left and right sides of the thoracic and lumbar spine [25]. While performing functional movements, upper body parts, like the shoulder, thoracic spine, and pelvis, are also part of the kinetic chain, and they involve a synergy of the multi-link kinetic chain [26]. Moreover, Sciascia, Thigpen [27] concluded that, for optimal movement function efficiency, problems with tightness of the leg, pelvic core, or scapular musculature, that is, a particular segment dysfunction, can result in an injury at the more distal segment. For example, shoulder injuries can occur in the kinetic chain due to too much overhead activity. However, for the LSS group in this study, although players provided a greater peak ground reaction force with the lead leg, they failed to generate faster ball velocity, showing that they were providing a greater reaction force to compensate for the low energy transfer efficiency in their kinetic chain. Therefore, the deep squat can be used to evaluate athletes, and it can assist athletes during training to improve their performance.

Although there was a significant difference in the peak GRF only in the lead leg between the HSS and the LSS, no significant difference was detected in the peak GRF time between the two groups. Interestingly, even with no notable disparity in ball velocity, the LSS group exhibited higher vertical ground reaction force in the lead leg. This unexpected finding diverges from earlier research by Kageyama, Sugiyama [9] which demonstrated that pitchers achieving high ball velocity typically exhibit enhanced ground reaction force (GRF) components in both the forward and vertical directions, particularly in the leading leg. Consequently, this study offers direct evidence that a larger propulsion force may not grant significant benefits if lower limb mobility in functional squat movements is restricted. Since the two groups in this study generated the same ball velocity, we can infer that baseball players who had been receiving long periods of specialized training for specific throwing movements adopted compensatory movements to maintain their athletic performance at a similar level. Changing the throwing movement might be the compensatory physiological response to maintaining or increasing the ball velocity, while the compensatory throwing mechanics may increase stress on the soft tissues, thereby affecting the magnitude, rate, and frequency of loading and altering the joint responsiveness [28]. Therefore, we speculate that players can maintain the same performance due to higher propulsion in long-term training. However, compensatory movements may increase the risk of injuries for baseball players who perform unilateral strength exercises for extended periods of time.

In this study, a thorough examination of the resultant impulse and its components in two distinct groups of pitchers demonstrated the time integration with the GRF during foot contact. We showed that, even with no notable disparity in ball velocity, the LSS group exhibited higher ground reaction impulse in both the pivot and lead leg. This unexpected finding stands in contrast to earlier research that often showcased high-velocity pitchers displaying greater resultant GRFs, forward force components, and vertical force components [9]. It may be attributed to the fact that all participants in both groups were elite athletes, ensuring a similar performance level. Consequently, this influence is reflected in lower efficiency, requiring a greater impulse for delivery. Furthermore, the leading leg of the LSS group exhibits lower efficiency and a greater impulse not only in the vertical but also in the forward component, whereas the pivot leg only demonstrates a greater vertical component. The results of this study offer deeper insights into the conventional understanding of the role of the legs and align well with other research that underscores the critical role of the lower limbs in establishing a stable base for efficient and safe arm motion generation, while also contributing to rotational momentum [29]. The HSS group, distinguished by enhanced lower limb mobility, may demonstrate superior proficiency in effectively coordinating trunk and pelvic rotation, utilizing similar lower limb mobility to distribute load across various body segments and transmit generated force.

The observed challenges in transferring propulsion force to the distal upper limb, thus achieving a similar athletic performance in the LSS group, could significantly impact long-term performance. Addressing these challenges through targeted training and interventions becomes imperative to enhance energy transfer efficiency, optimize athletic performance, and mitigate potential long-term effects on athletes within the LSS group. This approach is crucial for the overall development and sustained success of these athletes.

From the results of this study, it was observed that the LSS group exhibited a significantly higher vertical GRF in the lead leg, and the HSS group showed significantly higher efficiency in both peak force and impulse in the vertical GRF direction. This implies that the LSS group needs to generate more substantial momentum to achieve the same ball velocity as the HSS group. The HSS group’s utilization of lower limb ground reaction force for throwing was more efficient than that of the LSS group. Previous research has also indicated that improving pitching efficiency does not necessarily lead to a reduction in joint torque. However, enhancing efficiency can potentially increase ball velocity without increasing joint torque [12]. Therefore, baseball players with better lower limb mobility can more effectively apply ground reaction forces from their lower limbs during throwing and pitching.

Our study showed that the LSS group had a greater vertical GRF but was incapable of effectively increasing the ball speed over that of the HSS group. Previous studies have shown that, when stabilizing the player’s forward momentum when the leading foot touches the ground and converting the force into rotation, a large ground reaction force is required to support the push-off of the pivot leg and the braking of the leading leg [13]. Therefore, it is suggested that poor squat quality may affect the stability of the player’s forward momentum and the force transferred from the braked leading leg to the upper body, thereby reducing propulsion efficiency. Furthermore, it has been shown that the efficiency of energy transfer is critical for improving throwing performance and reducing the risk of injury due to overuse. It is important for players to reduce fatigue during long-term training. Minimizing the accumulation of injuries and increasing performance are critical factors in becoming a great athlete.

Regarding lower limb kinematics, the LSS and the HSS groups showed no significant differences in the maximum angle, minimum angle, or maximum angular displacement of hip flexion, knee extension, and ankle dorsiflexion (Table 4). The throwing motion is accomplished by transferring energy from the lower limbs to the upper limbs until the ball leaves the player’s hand. Therefore, we concluded that, without changing the range of movements, a compensatory propulsion force may have been applied to ensure a certain level of performance. This is unfavorable for athletes who must perform highly repetitive throwing motions, as the increased range of throwing motion may increase the stress on their muscles and joints. Baseball involves repetitive throwing motions, resulting in fatigue which may hinder joint stability and neuromuscular response; therefore, the performance of athletes may decrease, and joint instability and injuries can occur [30]. In conclusion, no significant differences were observed in the range of throwing motion in the lower limbs.

This study provides the first direct evidence that poor FMS squat performance and compensation movement lead to inefficiencies in kinetic chain performance in throwing, even though there was no difference in ball velocity between the groups since both groups are composed of athletes who are in elite baseball teams. Moreover, an athlete with poor FMS squat performance requires a potentially higher peak force to maintain a similar level of ball velocity, which results in a higher injury risk at maximum propulsion. However, in this study, due to the smaller range of throwing motion compared to the quality of squatting, the flexibility of the squatting functional motion does not lead to changes in the range of throwing motion and the maximum angular displacement of the lower limbs, but it might be caused by dynamic coordination, such as joint angle-acceleration coordination. Furthermore, in this study, FMS squats can serve as an effective evaluation tool for the performance efficiency of throwing movements on the transfer of ground reaction force to ball speed. They can also assist in evaluating the risk of injuries during propulsion movements and provide a reference for strength training for propulsion impact.

A major limitation of the study was that the performances of the participants were captured in a laboratory rather than a competitive setting. In other words, the setting was not on the field. A second limitation was that the throwing distance was limited to 7.5 m due to the constraints of the laboratory, and the participants could only be asked to simulate the actions of throwing on an actual field.

## 5. Conclusions

FMS squat scores can influence the GRF during throwing and may lead to abnormal compensations, thereby increasing the risk of injury, though the FMS squat scores were not associated with the range of motion of the lower limbs during throwing, or with throwing performance directly. The current evidence suggests that FMS squat performance is not only an assessment of lower extremity function quality but also a useful tool for detecting the efficiency and quality of energy transmission in the lower extremities during the throwing movement. This information may provide coaches and trainers with training programs and goals to consider in return-to-play strategies. Additionally, improving squat quality enhances propulsion efficiency and can be beneficial in training. This screening method can also help assess the risk of injury during propulsion activities and provide useful information for training guidelines.

## Figures and Tables

**Table 1:** Peak GRF and efficiency of throwing motion between HSS and LSS for both legs.

Valuables	HSS (N = 13) Mean ± SD	LSS (N = 9) Mean ± SD	*p*	ES
Pivot leg				
Peak Fx (%BW)	44.82 ± 11.53	41.73 ± 9.55	0.526	0.14
Peak Fz (%BW)	119.58 ± 8.78	121.56 ± 11.92	0.764	0.06
Velocity/Peak Fx (_m/s/N_)	0.71 ± 0.15	0.73 ± 0.11	0.764	0.06
Velocity/Peak Fz (_m/s/N_)	0.26 ± 0.03	0.24 ± 0.02	0.243	0.25
Leading leg				
Peak Fx (%BW)	−65.57 ± 12.61	−67.38 ± 10.93	0.570	0.12
Peak Fz (%BW)	158.81 ± 20.33	188.47 ± 35.51	0.030 *	0.46
Velocity/Peak Fx (_m/s/N_)	−0.48 ± 0.08	−0.45 ± 0.06	0.271	0.23
Velocity/Peak Fz (_m/s/N_)	0.19 ± 0.02	0.16 ± 0.02	0.002 *	0.66

ES = effect size * Significant at *p* < 0.05.

**Table 2:** Time-to-peak of GRF of throwing motion between HSS and LSS groups for both legs.

Time	HSS (N = 13) Mean ± SD	LSS (N = 9) Mean ± SD	*p*	ES
Pivot leg				
Time to Peak Fx (% of Throw)	87.08 ± 11.17	84.44 ± 14.18	0.570	0.12
Time to Peak Fz (% of Throw)	61.53 ± 20.36	55.79 ± 16.10	0.443	0.16
Leading leg				
Time to Peak Fx (% of Throw)	72.37 ± 14.61	71.79 ± 11.29	0.920	0.02
Time to Peak Fz (% of Throw)	81.81 ± 13.01	80.55 ± 7.51	0.973	0.01

ES = effect size.

**Table 3:** Impulse component and efficiency of throwing motion between HSS and LSS groups for both legs.

Variables	HSS (N = 13) Mean ± SD	LSS (N = 9) Mean ± SD	*p*	ES
Ball Velocity (m/s)	30.64 ± 3.33	29.56 ± 2.60	0.349	0.20
Pivot leg				
ImpulseF_resultant_ (N s)	54.50 ± 11.25	73.82 ± 22.60	0.035 *	0.45
ImpulseFx (N s)	14.02 ± 3.08	16.61 ± 4.51	0.049 *	0.42
ImpulseFz (N s)	52.58 ± 11.21	71.75 ± 22.67	0.048 *	0.42
Velocity/ImpulseF_resultant_ (_m/s/N_)	0.60 ± 0.19	0.43 ± 0.12	0.035 *	0.45
Velocity/Impulse Fx (_m/s/N_)	2.26 ± 0.48	1.86 ± 0.33	0.053	0.41
Velocity/Impulse Fz (_m/s/N_)	0.62 ± 0.21	0.45 ± 0.13	0.032 *	0.46
Leading leg				
ImpulseF_resultant_ (N s)	20.39 ± 5.04	31.27 ± 7.94	0.001 *	0.69
Impulse Fx (N s)	−7.76 ± 1.70	−11.37 ± 3.67	0.007 *	0.58
Impulse Fz (N s)	18.78 ± 4.83	29.04 ± 7.16	0.001 *	0.70
Velocity/ImpulseF_resultant_ (_m/s/N_)	1.64 ± 0.63	0.99 ± 0.21	0.003 *	0.63
Velocity/Impulse Fx (_m/s/N_)	−4.23 ± 1.45	−2.81 ± 0.77	0.005 *	0.60
Velocity/Impulse Fz (_m/s/N_)	1.79 ± 0.70	1.06 ± 0.23	0.021 *	0.49

ES = effect size * Significant at *p* < 0.05.

**Table 4:** The comparison of angular kinematics of lower extremity during throwing between HSS and LSS groups.

	Angle (deg)	HSS (N = 13) Mean ± SD	LSS (N = 9) Mean ± SD	*p*	ES
Hip Flexion	Maximum	153.17 ± 12.18	157.48 ± 11.75	0.483	0.15
	Minimum	124.05 ± 13.95	120.50 ± 10.40	0.616	0.11
	Displacement	29.12 ± 6.02	36.97 ± 13.74	0.217	0.26
Knee Extension	Maximum	173.59 ± 9.00	174.20 ± 6.17	0.973	0.01
	Minimum	128.15 ± 13.47	126.59 ± 15.07	0.815	0.05
	Displacement	45.44 ± 13.62	47.60 ± 15.24	0.815	0.05
Ankle Dorsiflexion	Maximum	118.53 ± 6.78	116.45 ± 6.71	0.404	0.18
	Minimum	100.12 ± 6.27	96.94 ± 4.23	0.102	0.35
	Displacement	18.41 ± 4.65	19.51 ± 5.29	0.616	0.11

ES = effect size value.

## Data Availability

All data are contained within the manuscript.
